# The Role of Emotional Skills (Competence) and Coping Strategies in Adolescent Depression

**DOI:** 10.3390/ejihpe13030041

**Published:** 2023-02-24

**Authors:** Dario Vucenovic, Gabriela Sipek, Katarina Jelic

**Affiliations:** 1 Faculty of Croatian Studies, University of Zagreb, 10 000 Zagreb, Croatia; 2 MIND Solutions d.o.o., 10 000 Zagreb, Croatia

**Keywords:** emotional skills, emotional competence, coping strategies, depression, adolescents

## Abstract

Depression is a state of low mood that can lead to several negative outcomes on thoughts, emotions, behaviors, and even physical state. With that in mind, it is important to detect individuals at risk of developing depressive symptoms early and identify protective factors. During the COVID-19 pandemic, adolescents emerged as one of the most vulnerable groups, with deteriorated anxiety and depression due to imposed social isolation, reduced social activities, and concerns over household status, health, and peer support. Distance learning through public service broadcasts and online tools lasted for several months, posing the need for adjustment. This study aimed to assess emotional competence and coping styles as predictors of depression in a sample of adolescents. The study was conducted in-person on a sample of 142 high school students. A high percentage of participants reported above-average levels of depression (21.1% severely depressed). On average, girls reported higher levels of depression than boys (t = 3.86, *p* < 0.01). Gender differences were also found in emotion-focused coping and avoidance, with girls scoring higher on both (*p* < 0.05). However, there were no gender differences in problem-focused coping or emotional competence. Hierarchical regression analysis concluded that perceiving and understanding emotions, expressing and naming emotions, regulating emotions, and avoidance were significant predictors of depression. This regression model explained 53% of depression variance, with the regulation of emotions being the most powerful predictor (*p* < 0.01). No mediating effect of coping styles on the relationship between emotional competence and depression was found in this study.

## 1. Introduction

Depression has steadily risen in the last few decades, affecting almost all age groups with early onset. It has become a global issue for youth mental health, especially after the pandemic. The COVID-19 pandemic severely threatened adolescents who faced imposed restrictions, changes in educational and leisure practices, increased digital activity [1], and disrupted interpersonal relations. According to the European Commission report [2], European adolescents’ mental health deteriorated before the COVID-19 pandemic. However, during the pandemic, health scares, loneliness, and tension made them fear for their future even more, and no other age group was affected like youth. Children and adolescents appear to be the most vulnerable group, after the chronically ill or elderly population, since they report deteriorated anxiety and depression levels after the pandemic [3]. Some data claim that almost 80% of children were affected negatively by the pandemic conditions [4]. A recent Canadian study showed how adolescent depression increased in general but increased even more within a female group [5]. Social isolation-imposed changes such as loneliness and remote and virtual learning were indicated. Reduced activities disabled valuable environmental protective factors such as social connection and support, which are some of the main community-level resilience factors for youth exposed to childhood adversity [6]. In Croatia, the first wave of restrictions was introduced on March 16. Until the end of May, distance learning was primarily delivered through public service broadcasts, online learning platforms, and tools. On March 19, the government-imposed restrictions on sports, culture, and religious and public gatherings. After that, the capital of Zagreb was hit by an earthquake, travelling between cities was banned, and remote working was encouraged wherever possible.

Adolescent depression manifests similarly to adults, with consistently low mood, hopelessness, and disinterest in activities being the predominant symptoms. However, in addition to lowered mood and increased dissatisfaction, adolescents tend to be angrier and more irritable [7]. Such behavior can be mistaken for antisocial or acting-out tendencies. Due to the growing prevalence of these symptoms and the fact that they are associated with an increased risk of reduced quality of cognitive, social, academic, and personal life [8], a more comprehensive understanding of this issue is necessary. Considering its early onset and how strongly symptoms of depression in adolescence predict an episode of major depression in adulthood [9], research efforts are needed to identify individuals at risk and protective factors, both personal and environmental.

Adolescence might be the optimal period for prevention and developing healthy habits for a lifetime [10]. Recent research highlights the role of emotional regulation [11] and coping strategies [12]. Emotional intelligence represents the ability to effectively process emotional information about oneself and others through emotional perception and using emotions to facilitate thinking, emotional understanding, and regulation [13]. A relationship between emotional intelligence and mental health has been found [14], and the mediating role of emotional regulation was emphasized [15]. That alone shows why adolescents with more advanced emotional intelligence express lower levels of depression. Emotion regulation skills develop through adolescence, and disrupted emotional regulation is a symptom of both anxiety and depression, as was found in self-report and neuroimaging measures [11].

A body of research focused on the relationship between coping styles and depression. Coping is generally defined as a psychological response to a stressful situation aimed at psychosocial adjustment. Lazarus and Folkman’s [16] stress and coping theory differentiate problem-focused and emotion-focused coping. According to this theory, problem-focused coping involves taking action to solve the problem and remove the stressor. In contrast, emotion-focused coping reduces negative emotions associated with the stressor. In addition, Endler and Parker [17] conceptualized avoidance as another coping style based on psychological or behavioral distraction from the source of stress. A strong connection exists between higher levels of depression and avoidance coping styles and lower levels of depression and problem-focused coping styles [18]. Some authors suggest that emotional intelligence affects depression by regulating coping styles [19]. There is a possibility that emotional intelligence influences the selection and implementation of coping strategies, making emotionally competent individuals more likely to choose strategies that lead to adjustment [19]. 

Higher levels of emotional intelligence, especially the ability to regulate emotions, are often associated with more frequent use of problem-focused coping [20]. Emotional intelligence affects the choice of coping style, which determines the level of depression. Moreover, higher levels of emotional intelligence affect coping effectiveness by enhancing the positive effect of active coping and minimizing the negative impact of avoidant coping on mental health [21]. 

Despite speculations about the interaction of emotional intelligence and coping styles in predicting depression, little research has empirically and systematically explored this relationship and all the involved mechanisms. This study examines the role of emotional competence and coping styles in predicting depression in adolescents. Based on previous research, we expect girls to achieve significantly higher results on depression and overall emotional competence and to express a strong preference for an emotion-focused coping style. Women tend to employ this style more often to manage stressors [22], and, inversely, those who use it report more depressive symptoms [23]. Gender, emotional intelligence, and coping styles are expected to significantly predict levels of depression, with coping styles as a mediator variable. Some authors [19] suggest that emotional intelligence affects depression by regulating coping styles, leading individuals to choose strategies that provide better outcomes, such as problem-solving or enhancing positive effects through social support and less avoidant tendencies. Therefore, we are examining how accurate the presented model is for adolescents in the post-COVID-19 era and whether emotional regulation protects them even in such distorted social circumstances. 

## 2. Materials and Methods

### 2.1. Participants

A total of 142 second-grade high school students from an urban area, living with both parents and with no chronic illness or cognitive impairment, participated in the research. Participants were introduced to the study details, and the researcher obtained their written informed consent. We wanted to control for any age-related effects and thus chose to narrow down the age group. Since school prevention programs in Croatia started in second grade, we tried to collect pre-prevention intervention data for implication purposes. Participants filled out an online survey during school hours to ensure the validity of obtained data. The age of the participants ranged between 14 and 17 years, with a mode value of 16 (108 participants) and a mean of 15.92 (SD = 0.53). Of the participants, 20 were 15-year-olds, and 14 were 17-year-olds. In total, 60% of them were females. No participants were excluded from the analysis 

### 2.2. Questionnaires

Gathered information from participants included: sociodemographic data (age, gender, grade), the Emotional Skills and Competence Questionnaire (ESCQ-45), a short version of the Coping Inventory for Adolescents, and the Depression, Anxiety and Stress Scale (DASS). All questionnaires were validated on a Croatian sample of adolescents. 

#### 2.2.1. The Emotional Skills and Competence Questionnaire (ESCQ-45)

The Emotional Skills and Competence Questionnaire (ESCQ-45) [24] was used to assess emotional intelligence. The questionnaire consists of 45 items divided into three subscales: the ability to perceive and understand emotions (15 items), the ability to express and name emotions (14 items) and the ability to manage emotions (16 items). The participant’s task is to assess the extent to which a particular item applies to them on a Likert-type scale (1–5). The overall score is formed by dividing the sum of the results by the number of items, where a higher score implies a higher level of emotional competence. The reliability of the total score varies from α = 0.87 to α = 0.93 in this research, similar to the original author’s findings.

#### 2.2.2. Short Version of the Coping Inventory for Adolescents

A short version of the Coping Inventory for Adolescents [25] was used to determine participants’ coping styles. It consists of 18 items divided into 3 subscales: Problem-focused coping, emotion-focused coping, and avoidance. The participant’s task is to assess how often they use a particular coping strategy on a Likert-type scale (1–4). Each subscale forms into total sums, where a higher result means a greater tendency to use that coping style. According to the authors, each subscale has adequate reliability, with avoidance being the lowest. In our research, avoidance was the most reliable (α = 0.88), next to the problem-focused (α = 0.74) and the emotion-focused coping subscale (α = 0.65).

#### 2.2.3. Depression Anxiety Stress Scale (DASS)

The Depression Anxiety Stress Scale (DASS) was used to assess the levels of depression in adolescents [26]. The scale consists of 42 items divided into three subscales: Depression, anxiety and stress (14 items each), and we used the depression subscale. Participants estimate how often they experienced specific symptoms in the last week on a Likert-type scale (0–3). The reliability for the depression subscale in Croatian validation was α = 0.85, and in this research, it was α = 0.96.

### 2.3. Procedure

The study was conducted following the Declaration of Helsinki and approved by the University of Zagreb, Faculty of Croatian Studies (protocol code 380-58/506-22-0001). The research was conducted during the second wave of the COVID-19 pandemic and after the lockdown, and schools were no longer practising online classes. After the methodological and ethical approval, schools were contacted to grant classroom access. Principals and staff were given detailed instructions about the research, following the Croatian Code of Ethics for Research with Children and parental agreements for school extracurricular activities. Parents were notified about the entire procedure in advance, and all of them gave informed consent. Students were informed about the research’s aims, granted anonymity, and signed the informed consent. After filling out the questionnaire, they were given a short educational brochure about mental health, including a list of adolescent depression symptoms and free public mental health practitioners in their school area.

### 2.4. Data Analysis

All analyses were made utilizing IBM^®^ SPSS^®^ Statistics version 29. To determine the reliability of chosen questionnaires, we used Cronbach’s alpha coefficient. Descriptive analysis included the Kolmogorov–Smirnov goodness-of-fit test and Levene’s homogeneity of variance test to ensure parametric preconditions. A T-test for independent samples (Cohen’s D statistic) was used to estimate between-subjects effects. Pearson’s correlation coefficient was used for all variables measured on pseudo-interval Likert scales as a base for hierarchical (linear) regression analysis and mediation analysis with the SPSS PROCESS macro developed by Andrew Hayes (Model 1).

## 3. Results

### 3.1. Descriptive Data

We found no extreme results according to the z-values of individual results, as Field and Miles [27] suggested. The next step was testing the parametric procedures’ assumptions, as presented in Table 1.

The Kolmogorov–Smirnov goodness-of-fit test indicated that the abilities to perceive and understand emotions and manage them fit within a normal distribution range. At the same time, all other variables significantly deviated from it. Since the asymmetry values of the distribution in this sample did not exceed 0.51, and the flattening values did not exceed 0.88 (see Table 1), the distributions were considered normal for parametric procedures [28]. According to the DASS norms, most participants reported average levels of depression (37.3%), while 21.1% suffered from extremely severe depression. Mild, medium, and severe depression levels were evenly distributed at around 13–14% of our sample.

### 3.2. Gender Differences in Depression, Emotional Competence, and Coping Styles

A *T*-test for independent samples was conducted to determine gender differences in the observed variables. Cohen’s D statistic confirmed that, as expected, girls reported significantly higher levels of depression (d = 0.99), but there were no differences in self-assessed emotional competence and problem-focused coping style. Results also suggest gender differences in emotion-focused coping (d = 0.47) and avoidance (d = 0.59) styles, which are more common for adolescent girls, as is shown in Table 2.

### 3.3. The Relationship between Depression, Emotional Competence, and Coping Styles

Our first step was calculating a correlation matrix and Pearson’s correlation coefficient for all variables measured on pseudo-interval Likert scales to determine whether selected predictors correlate with the criteria variable, i.e., depression. As presented in Table 3, all variables except for the ability to perceive and understand emotions correlate significantly. Between them, emotional competencies were more strongly associated with depression than coping styles, and participants with higher emotional competencies reported lower levels of depression. Additionally, emotion- and problem-oriented strategies were negatively associated with depression, as opposed to avoidance style.

Emotional competence was significantly related to problem-focused and emotion-focused coping but not to avoidance style. As expected, a problem-focused coping style was strongly associated with the ability to manage emotions through activities aimed at handling stressful situations or eliminating their sources, and emotion-focused coping showed the highest correlation with the strategy of naming and expressing emotions. Furthermore, Table 4 shows the results of hierarchical regression analysis to determine the independent and overall predictive value of gender, coping styles, and emotional competence in predicting depression.

**Figure 1:**
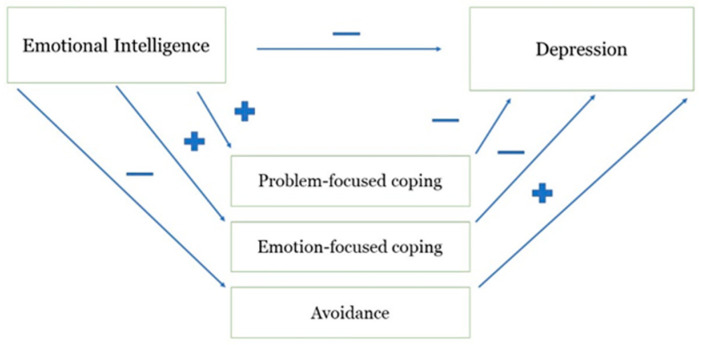
Hypothesized path values for mediation model where coping styles mediate the effect of emotional competence on depression.

Given the established gender differences in depression, gender was used as the first independent predictor, but it covers only 9% of the variance in depressive symptoms. Coping styles explained an additional 22% in the next step, the same as emotional competence in the third and final steps. Overall, our model explained 53% of criteria variance and confirmed the hypothesized model of emotional intelligence and well-being [29], where coping strategies represent mediator variables between EI and mental health outcomes (Figure 1). Since correlation assumptions for that specific model are proven in our sample, we proceeded with the mediation analysis steps [30] (see Table 5 and Figure 1) using the PROCESS macro developed by Andrew Hayes [31], Model 1.

We proceeded with mediation analysis [30] and first checked the relationship between the predictor and the criterion variable, which was significant (β = −0.44). The relationship between the predictor (emotional competence) and the potential mediator (coping styles) proved to be significant, except for the avoidance style. Therefore, avoidance was not included in the third step, where coping styles did not predict levels of depression after controlling for emotional competence, meaning we do not have enough evidence to make a conclusion about the presence of a mediating effect of coping styles.

## 4. Discussion

The main goal of this research was to determine whether the assumed model [19] of gender, emotional competence, and coping style can predict depression levels in adolescents. Descriptive data showed that 63% of participants reported above-average or subsyndromal levels of depression, suggesting adolescents were experiencing clinical symptoms shortly after the second wave of the COVID-19 pandemic. This is in line with previous research stating that between 20 and 50% of adolescents experience symptoms of depression [32]. The global prevalence rate of self-reported depressive symptoms among adolescents increased from 24% to 37% in 2001–2010 [33], and recent data suggest another decline in their general resilience and coping abilities.

### 4.1. Gender Differences in Depression, Emotional Competence, and Coping Styles

Previous findings [34] imply that gender differences in depression become noticeable during puberty. The combined effect of biological factors, multiple changes in self-image, and identity confusion can trigger negative affect, avoidance, and helplessness [35]. On average, depression rises for female adolescents and remains relatively stable for boys [34]. Changes in puberty are only partially responsible. Other factors, such as negative cognitive style, social pressure, and a higher frequency of stressful events, can contribute to the increased risk of depression in adolescent girls [36]. Our findings support similar post-pandemic reports where female adolescents were more vulnerable [5].

Regarding gender differences in levels of emotional competence, some research found none [37], while others suggest that men tend to achieve higher levels on self-report measures, while women perform better on objective measures, e.g., tests [38]. In addition, emotional competence is not a one-dimensional construct, and some researchers state that women develop a more remarkable ability to perceive emotions. At the same time, men are better at managing emotions, perhaps due to better down-regulation of amygdala activity combined with less prefrontal activity during regulation, making them more effective in regulating negative emotions [39]. While no significant gender differences were found in problem-focused coping, the emotion-focused and avoidance styles were more frequent in girls. We could argue that the avoidance style is more commonly found in clinical groups regardless of gender, as it was found that immature defense styles and avoidance coping mediated psychiatric symptomatology after victimization [40]. Another viewpoint derives from social role theory, where social behavior tends to be affected by widely shared gender stereotypes, one of which is the tendency to express feelings openly, which could explain why females are more inclined to use emotion-based coping strategies and social support-seeking. 

Women tend to use social support more often in stressful situations, and items from that specific subscale partially measure social support seeking. Regarding problem-oriented coping, previous research is not consistent, but it seems that, on average, men (or those high in masculinity) use problem-focused coping strategies more often [41]. It appears that gender affects both perceptions of a situation as stressful (or not) and the coping response or behaviors and their consequences [42]. However, results indicating a greater tendency toward the avoidance style in women should be interpreted cautiously. Although many other authors agree with this outcome [43], others point out that men prefer avoidance strategies [44] to compass emotional distance, which can help reduce stress short-term through distraction and denial. These inconsistencies may also reflect differences in theoretical operationalizations of coping mechanisms, stress, and general vulnerability.

The bivariate correlation analysis results indicated a moderate to high correlation between emotional competence and depression, as expected in previous reports [15]. Total ESCQ-45 score and naming and managing emotions were negatively related to depression, in accordance with the rest of the empirical data [45]. Emotion-focused coping showed the highest correlation with the strategy of naming and expressing emotions since they tend to help individuals to diminish the emotional consequences of stressful events, such as expressing emotions destructively or inappropriately [46] and can help verbalize internal mental health status when seeking help or support. The ability to perceive and understand emotions was often positively linked to depression [38]. Understanding emotions is a necessary first step in expressing and managing them. However, prolonged focus on emotions can lead to rumination, which triggers negative emotions and depressive symptoms [47]. For those reasons, recognizing and naming personal and other people’s emotional states is an advantage for favorable outcomes only in low-stress environments or combined with high emotional regulation. In another way, it can make youth more prone to experiencing guilt, shame, anger, inadequacy, fear, and loneliness.

On average, participants with a more problem-focused [19] and emotion-focused coping style had lower levels of depression, as was hypothesized based on comparative research [48,49]. Avoidance was negatively linked to depression, as suggested by some authors [18]. Focus on emotions proved to be an adaptive strategy for adolescents. Based on this questionnaire, emotion-focused coping represents behaviors such as seeking social support and openly expressing emotions. Perhaps emotion- and problem-oriented coping are not necessarily opposing strategies. Rather than being opposites, when combined they tend to lead to more favorable outcomes such as psychological adaptation [50].

Emotional competence and stress coping styles did not correlate entirely. Emotionally competent adolescents preferred problem- and emotion-focused coping styles more than avoidant ones. Emotional intelligence or emotional knowledge allows us to recognize, select, and use more adaptive strategies in stressful situations [20,21]. Effective coping with pressure, conflict, and interpersonal relations is an essential trait of emotionally intelligent individuals [51]. They tend to modify their environments to reduce frustration and negative emotions, monitor emotional cues and learn from social situations faster so they do not have to use avoidance as a coping style [52]. Regulating emotions improves with age, when prefrontal brain regions mature and our cognitive control and executive functioning develop. That makes us successful in complex problem-solving, visualizing different outcomes, and becoming less reactive to external stimuli [53].

### 4.2. The Relationship between Depression, Emotional Competence, and Coping Styles

The linear hierarchical regression analysis managed to predict depression in adolescents. Gender and problem- and emotion-focused coping ceased to be significant predictors of depression when emotional competence entered analysis, as expected [14,48]. 

Only avoidance remained a significant predictor of depression, and emotional competence did not affect avoidance in mediation analysis. Avoidance contributes to the maintenance of depressive symptoms by delaying the elimination of the source of stress [54]. Similarly, higher emotional intelligence helps maintain a stable mood after stressful situations, and thus leads to better adaptation [21]. Finally, some [55] believe that control over rumination is key to emotional adaptation. An emotionally competent person will choose a more adaptive coping strategy [19,56].

The mediating influence of stress coping styles on the relationship between emotional competence and depression should have been verified. However, theoretical assumptions for mediation were not achieved. Although authors assume that emotional intelligence mitigates the negative impact of stress through selecting an adaptive coping strategy and thus contributes to the preservation of mental health [56], only a few studies have examined that [19]. Emotional intelligence could determine the choice of coping style, affecting depression levels. A notable difference in research results might be the consequence of the decision to use an emotional intelligence test instead of self-report measures [19]. Combined with zero evidence for gender differences in emotional competence, we conclude that subjective measures may not be the best option for the adolescent population or should be combined with more objective instruments.

### 4.3. Implications and Limitations

The results of this research represent valuable data about depression prevalence and preferred coping styles in the Croatian youth population. They should be used primarily as guidelines for future research and efforts to incorporate emotional literacy in school programs in a way that promotes mental health and prolongs the onset of depression, but that also serves as a tool for self-monitoring programs aimed at developing socio-emotional skills in children, and adolescents have repeatedly proven to be efficient in improving their mental health and life satisfaction. Programs aimed at developing emotional competence can significantly improve adolescents’ mental health six months after the program [57].

The research was conducted on a small, non-random, convenient sample of adolescents from only four high schools, reducing the external validity. Since the total population of high school students in this city is around 35.000, the sample size should aim at a minimum of 1–2% of the population (sample size calculation indicates at least 380 participants or, as some suggest, over 250 to stabilize correlations [58]). However, most schools were not allowing paper surveys and were still too concerned over coronavirus transmission, but for the sake of internal validity, we opted for an in vivo approach at the expense of sample size. It is possible that, based on some systematic factor, some students were more inclined to participate in the research, contributing to higher average levels of depression. Self-reports are traditionally loaded with several issues but remain a gold standard for correlation research. Some additional predictor variables should be incorporated into a comprehensive, longitudinal study. Perhaps controlling for five broad personality traits would elucidate which individuals faced the most negative outcomes during the pandemic. Several studies inspected differences in personality and cognitive skills. They found that individuals high on openness and extraversion during the pandemic suffered more, while those higher in agreeableness were less affected. In the same research, specific cognitive skills presented risk factors for females [59]. Another key limitation represents possible unmeasured confounders or spurious associations related to coping styles and mental health, including physical health. In a more recent paper, emotional ability decreased with age and lower income [60]. Education, recent income declines, and poor housing were found to be associated with a higher prevalence of mental disorders in adults [61]. In general, we excluded family-related variables that might have an impact, such as parental education level, heredity of depression, etc.

To conclude, the COVID-19 pandemic posed a significant threat to youths’ mental health and was possibly mediated by coping styles, including behavior patterns such as increased digital activity, physical activities, or even substance abuse during the lockdown period. Young people, women, and minorities use social networks more often [62], and while they are an important outlet for socializing, education, and fun, they can also indicate avoidance strategies or entice social comparison, depression, fear of missing out, etc. It is reported that students who used electronic devices after 10:00 pm during the last 6 months had poorer academic performance and sleep quality [63]. Some might suggest that, during the pandemic, adolescent substance use increased due to stress, social isolation, and even boredom [64]. Worrying about a potential new wave of coronavirus could affect the adolescent’s ability to experience positive emotions and trigger negative behavior patterns during (partial) social isolation. 

## 5. Conclusions

The obtained results indicate that some adolescents in Croatia experience severe and extremely severe symptoms of depression, predominantly girls who also tend to use avoidance strategies more often, perhaps due to a lack of social support or because their social support system is ineffective in mitigating stress.

Emotional competence could predict depression symptoms, along with avoidance strategies. We could theorize how the COVID-19 pandemic induced adolescents’ tendencies to avoid unpleasant emotions, interpersonal conflicts, financial difficulties, or any other major repercussions of a lockdown. We could see how, for example, online schooling, academic burnout, and social isolation could affect vulnerable youth. Even though our test model explained 53% of the variance in depression, with the ability to manage emotions being the most significant predictor, there was no evidence supporting the hypothesized mediating effect of coping styles, suggesting their effects are independent, but also that emotional regulation is key concept to be explored and implemented in the school curriculum, such as RULER [30,65].

## Figures and Tables

**Table 1:** Descriptive data for all observed variables (*N* = 142).

					Asymmetry		
		M	SD	TR	S	K	K-S
Depression		16.10	12.24	0–42	0.51	−0.88	0.11 **
ESCQ-45	ESCQ-45 total score	3.43	0.53	1–5	0.44	0.15	0.08 *
	Perceiving and understanding emotions	3.62	0.69	1–5	−0.10	−0.29	0.05
	Expressing and naming emotions	3.05	0.75	1–5	0.26	−0.36	0.09 *
	Managing emotions	3.58	0.53	1–5	0.11	−0.05	0.05
Coping style	Problem-focused coping	15.82	3.83	0–24	−0.26	−0.46	0.09 *
	Emotion-focused coping	11.06	5.62	0–24	−0.03	−0.59	0.08 *
	Avoidance	13.75	4.40	0–24	0.12	−0.75	0.12 **

* *p* < 0.05; ** *p* < 0.01; S-Skewness; K-Kurtosis; KS = Kolmogorov–Smirnov Goodness-of-Fit test.

**Table 2:** Results of T-test for independent samples on all observed variables.

	Girls (*N* = 85)		Boys (*N* = 57)		
	M	SD	M	SD	T
Depression	19.09	12.51	11.63	10.42	3.86 **
ESCQ-45 total score	3.41	0.52	3.45	0.56	−0.45
Perceiving and understanding emotions	3.71	0.70	3.49	0.65	1.87
Expressing and naming emotions	2.96	0.76	3.19	0.73	−1.84
Managing emotions	3.53	0.50	3.65	0.58	−1.26
Problem-focused coping	15.73	3.81	15.95	3.89	−0.33
Emotion-focused coping	12.09	5.56	9.51	5.41	2.75 *
Avoidance	14.75	4.27	12.26	4.19	3.43 *

* *p* < 0.05; ** *p* < 0.01.

**Table 3:** Pearson correlation matrix for predictor and criteria variable (N = 142).

		1.	2.	3.	4.	5.	6.	7.	8.
1.	Depression	1							
2.	ESCQ-45 PUE	−0.04	1						
3.	ESCQ-45 ENE	−0.52 **	0.38 **	1					
4.	ESCQ-45 ME	−0.55 **	0.51 **	0.63 **	1				
5.	ESCQ-45 total score	−0.44 **	0.78 **	0.82 **	0.85 **	1			
6.	Problem-focused coping	−0.34 **	0.40 **	0.35 **	0.52 **	0.51 **	1		
7.	Emotion-focused coping	−0.23 **	0.17 *	0.46 **	0.25 **	0.36 **	0.29 **	1	
8.	Avoidance	0.39 **	0.06	−0.21 *	−0.08	−0.10	−0.22 **	0.00	1

Notes: ESCQ-45 PUE = Perceiving and understanding emotions; ESCQ-45 ENE = Expressing and naming emotions; ESCQ-45 ME = Managing emotions; * *p* < 0.05; ** *p* < 0.01.

**Table 4:** Hierarchical regression analysis predicting depression levels based on gender, coping styles, and emotional competence.

	Β	R	R^2^	F	ΔR^2^	ΔF
*Step one*		0.30	0.09	13.84 **	0.09	13.84 **
Gender	−0.30 **					
*Step two*		0.56	0.31	15.62 **	0.22	14.84 **
Gender	−0.27 **					
Problem-focused coping	−0.21 *					
Emotion-focused coping	−0.24 *					
Avoidance	0.27 *					
*Step three*		0.73	0.53	21.56 **	0.22	20.55 **
Gender	−0.12					
Problem-focused coping	−0.05					
Emotion-focused coping	−0.09					
Avoidance	0.25 **					
Perceiving and understanding emotions	0.28 **					
Expressing and naming emotions	−0.20 *					
Managing emotions	−0.49 **					

* *p* < 0.05; ** *p* < 0.01.

**Table 5:** Mediation analysis: Do coping styles mediate the effect of emotional competence on depression?

	Β	t
*Step one*		
Emotional competence → Depression	−0.44	−5.85 **
*Step two*		
Emotional competence → Problem-focused coping	0.51	7.07 **
Emotional competence → Emotion-focused coping	0.36	4.63 **
Emotional competence → Avoidance	−0.10	−1.14
*Step three*		
Problem-focused coping → Depression	−0.16	−1.78
Emotion-focused coping → Depression	−0.08	−1.04

** *p* < 0.01.

## Data Availability

The data presented in this study are available on request from the corresponding author. The data are not publicly available due to lack of funding.

## References

[1] Kopilaš V., Hasratian A.M., Martinelli L., Ivkić G., Brajković L., Gajović S. (2021). Self-Perceived Mental Health Status, Digital Activity, and Physical Distancing in the Context of Lockdown Versus Not-in-Lockdown Measures in Italy and Croatia: Cross-Sectional Study in the Early Ascending Phase of the COVID-19 Pandemic in March 2020. Front. Psychol..

[2] European Education and Culture Executive Agency (2022). The Impact of the COVID-19 Pandemic on the Mental Health of Young People: Policy Responses in European Countries. https://data.europa.eu/doi/10.2797/299233.

[3] Wang S., Chen L., Ran H., Che Y., Fang D., Sun H., Peng J., Liang X., Xiao Y. (2022). Depression and anxiety among children and adolescents pre and post COVID-19: A comparative meta-analysis. Front. Psychiatry.

[4] Panda P.K., Gupta J., Chowdhury S.R., Kumar R., Meena A.K., Madaan P., Sharawat I.K., Gulati S. (2021). Psychological and Behavioral Impact of Lockdown and Quarantine Measures for COVID-19 Pandemic on Children, Adolescents and Caregivers: A Systematic Review and Meta-Analysis. J. Trop. Pediatr..

[5] Hollenstein T., Colasante T., Lougheed J.P. (2021). Adolescent and Maternal Anxiety Symptoms Decreased but Depressive Symptoms Increased before to during COVID-19 Lockdown. J. Res. Adolesc..

[6] Fritz J., de Graaff A.M., Caisley H., van Harmelen A.-L., Wilkinson P.O. (2018). A Systematic Review of Amenable Resilience Factors That Moderate and/or Mediate the Relationship between Childhood Adversity and Mental Health in Young People. Front. Psychiatry.

[7] American Psychiatric Association (2013). Diagnostic and Statistical Manual of Mental Disorders.

[8] Richards D. (2011). Prevalence and clinical course of depression: A review. Clin. Psychol. Rev..

[9] Pine D.S., Cohen E., Cohen P., Brook J. (1999). Adolescent Depressive Symptoms as Predictors of Adult Depression: Moodiness or Mood Disorder?. Am. J. Psychiatry.

[10] Gladstone T.R.G., Beardslee W.R., O’Connor E.E. (2011). The Prevention of Adolescent Depression. Psychiatr. Clin. N. Am..

[11] Young K., Sandman C., Craske M. (2019). Positive and Negative Emotion Regulation in Adolescence: Links to Anxiety and Depression. Brain Sci..

[12] Richardson C.E., Magson N.R., Fardouly J., Oar E.L., Forbes M.K., Johnco C.J., Rapee R.M. (2021). Longitudinal Associations between Coping Strategies and Psychopathology in Pre-adolescence. J. Youth Adolesc..

[13] Mayer J.D., Caruso D.R., Salovey P. (2016). The Ability Model of Emotional Intelligence: Principles and Updates. Emot. Rev..

[14] Martins A., Ramalho N., Morin E. (2010). A comprehensive meta-analysis of the relationship between Emotional Intelligence and health. Pers. Individ. Differ..

[15] Gomez-Baya D., Mendoza R., Paino S., de Matos M.G. (2017). Perceived emotional intelligence as a predictor of depressive symptoms during mid-adolescence: A two-year longitudinal study on gender differences. Pers. Individ. Differ..

[16] Lazarus R., Folkman S. (1984). Stress, Appraisal, and Coping.

[17] Endler N.S., Parker J.D. (1990). Multidimensional assessment of coping: A critical evaluation. J. Pers. Soc. Psychol..

[18] Aranda M.P., Castaneda I., Lee P.-J., Sobel E. (2001). Stress, social support, and coping as predictors of depressive symptoms: Gender differences among Mexican Americans. Soc. Work Res..

[19] Davis S.K., Humphrey N. (2012). The influence of emotional intelligence (EI) on coping and mental health in adolescence: Divergent roles for trait and ability EI. J. Adolesc..

[20] Petrides K., Pita R., Kokkinaki F. (2007). The location of trait emotional intelligence in personality factor space. Br. J. Psychol..

[21] Mikolajczak M., Nelis D., Hansenne M., Quoidbach J. (2008). If you can regulate sadness, you can probably regulate shame: Associations between trait emotional intelligence, emotion regulation and coping efficiency across discrete emotions. Pers. Individ. Differ..

[22] Mezulis A.H., Abramson L.Y., Hyde J.S. (2002). Domain Specificity of Gender Differences in Rumination. J. Cogn. Psychother..

[23] Bennett K.K., Compas B.E., Beckjord E., Glinder J.G. (2005). Self-Blame and Distress Among Women with Newly Diagnosed Breast Cancer. J. Behav. Med..

[24] Taksic V. (2002). Questionnaires of emotional intelligence (competence) UEK. Collection of Psychological Scales and Questionnaires.

[25] Krapic N., Kardum I. (2003). Stilovi suočavanja sa stresom kod adolescenata: Konstrukcija i validacija upitnika. Drus. Istraz..

[26] Lovibond P.F., Lovibond S.H. (1995). The structure of negative emotional states: Comparison of the Depression Anxiety Stress Scales (DASS) with the Beck Depression and Anxiety Inventories. Behav. Res. Ther..

[27] Field A., Miles J. (2010). Discovering Statistics Using SAS.

[28] Kline R. (2015). Principles and Practice of Structural Equation Modeling.

[29] MacCann C., Double K.S., Clarke I.E. (2022). Lower Avoidant Coping Mediates the Relationship of Emotional Intelligence with Well-Being and Ill-Being. Front. Psychol..

[30] Baron R.M., Kenny D.A. (1986). The moderator–mediator variable distinction in social psychological research: Conceptual, strategic, and statistical considerations. J. Pers. Soc. Psychol..

[31] Hayes A.F. (2022). Introduction to Mediation, Moderation, and Conditional Process Analysis: A Regression-Based Approach.

[32] Kessler R.C., Berglund P., Demler O., Jin R., Koretz D., Merikangas K.R., Rush A.J., Walters E.E., Wang P.S. (2003). The Epidemiology of Major Depressive Disorder. JAMA.

[33] Shorey S., Ng E.D., Wong C.H.J. (2022). Global prevalence of depression and elevated depressive symptoms among adolescents: A systematic review and meta-analysis. Br. J. Clin. Psychol..

[34] Galambos N.L., Leadbeater B.J., Barker E.T. (2004). Gender differences in and risk factors for depression in adolescence: A 4-year longitudinal study. Int. J. Behav. Dev..

[35] Twenge J.M., Nolen-Hoeksema S. (2002). Age, gender, race, socioeconomic status, and birth cohort difference on the children’s depression inventory: A meta-analysis. J. Abnorm. Psychol..

[36] Hyde J.S., Mezulis A.H. (2020). Gender Differences in Depression: Biological, Affective, Cognitive, and Sociocultural Factors. Harv. Rev. Psychiatry.

[37] Meshkat M., Nejati R. (2017). Does Emotional Intelligence Depend on Gender? A Study on Undergraduate English Majors of Three Iranian Universities. SAGE Open.

[38] Salguero J.M., Palomera R., Fernández-Berrocal P. (2012). Perceived emotional intelligence as predictor of psychological adjustment in adolescents: A 1-year prospective study. Eur. J. Psychol. Educ..

[39] McRae K., Ochsner K.N., Mauss I.B., Gabrieli J.J.D., Gross J.J. (2008). Gender Differences in Emotion Regulation: An fMRI Study of Cognitive Reappraisal. Group Process. Intergroup Relat..

[40] Zerach G., Elklit A. (2020). Polyvictimization and Psychological Distress in Early Adolescence: A Mediation Model of Defense Mechanisms and Coping Styles. J. Interpers. Violence.

[41] Dyson R., Renk K. (2006). Freshmen adaptation to university life: Depressive symptoms, stress, and coping. J. Clin. Psychol..

[42] Matud M.P. (2004). Gender differences in stress and coping styles. Pers. Individ. Differ..

[43] Griffith M.A., Dubow E.F., Ippolito M.F. (2000). Developmental and Cross-Situational Differences in Adolescents’ Coping Strategies. J. Youth Adolesc..

[44] Hampel P., Petermann F. (2005). Age and Gender Effects on Coping in Children and Adolescents. J. Youth Adolesc..

[45] Schutte N.S., Malouff J.M., Thorsteinsson E.B., Bhullar N., Rooke S.E. (2007). A meta-analytic investigation of the relationship between emotional intelligence and health. Pers. Individ. Differ..

[46] Schoenmakers E.C., van Tilburg T.G., Fokkema T. (2015). Problem-focused and emotion-focused coping options and loneliness: How are they related?. Eur. J. Ageing.

[47] Compare A., Zarbo C., Shonin E., van Gordon W., Marconi C. (2014). Emotional Regulation and Depression: A Potential Mediator between Heart and Mind. Cardiovasc. Psychiatry Neurol..

[48] Compas B.E., Connor-Smith J.K., Saltzman H., Thomsen A.H., Wadsworth M.E. (2001). Coping with stress during childhood and adolescence: Problems, progress, and potential in theory and research. Psychol. Bull..

[49] Howerton A., van Gundy K. (2009). Sex differences in coping styles and implications for depressed mood. Int. J. Stress Manag..

[50] Carver C.S., Scheier M.F. (1994). Situational coping and coping dispositions in a stressful transaction. J. Pers. Soc. Psychol..

[51] Bar-On R., Parker J.D.A. (2000). Emotional and social intelligence: Insights from the Emotional Quotient Inventory. The Handbook of Emotional Intelligence: Theory, Development, Assessment, and Application at Home, School, and in the Workplace.

[52] Wells A. (2000). Emotional Disorders and Metacognition: Innovative Cognitive Therapy.

[53] Martin R.E., Ochsner K.N. (2016). The neuroscience of emotion regulation development: Implications for education. Curr. Opin. Behav. Sci..

[54] Holahan C.J., Moos R.H., Holahan C.K., Brennan P.L., Schutte K.K. (2005). Stress Generation, Avoidance Coping, and Depressive Symptoms: A 10-Year Model. J. Consult. Clin. Psychol..

[55] Salovey P., Mayer J.D., Goldman S.L., Turvey C., Palfai T.P. (1995). Emotional attention, clarity, and repair: Exploring emotional intelligence using the Trait Meta-Mood Scale. Emotion, Disclosure, & Health.

[56] Keefer K., Parker J.D.A., Saklofske D.H. (2009). Emotional Intelligence and Physical Health. Assessing Emotional Intelligence.

[57] Ruiz-Aranda D., Castillo R., Salguero J.M., Cabello R., Fernández-Berrocal P., Balluerka N. (2012). Short- and Midterm Effects of Emotional Intelligence Training on Adolescent Mental Health. J. Adolesc. Health.

[58] Schönbrodt F.D., Perugini M. (2013). At what sample size do correlations stabilize?. J. Res. Pers..

[59] Proto E., Zhang A. (2021). COVID-19 and mental health of individuals with different personalities. Proc. Natl. Acad. Sci. USA.

[60] Alnjadat R., Al-Rawashdeh A. (2021). Confounding Factors Affecting the Emotional Intelligence Amongst Jordanian Nursing and Midwifery Undergraduate Students During the COVID-19 Pandemic’s Outbreak: A Cross-Sectional Study Using USMEQ-i. Front. Psychol..

[61] Araya R. (2003). Education and income: Which is more important for mental health?. J. Epidemiol. Community Health.

[62] Kircaburun K., Alhabash S., Tosuntaş Ş.B., Griffiths M.D. (2020). Uses and Gratifications of Problematic Social Media Use Among University Students: A Simultaneous Examination of the Big Five of Personality Traits, Social Media Platforms, and Social Media Use Motives. Int. J. Ment. Health Addict..

[63] Mao Y., Xie B., Chen B., Cai Y., Wu J., Zhang J., Shao R., Li Y. (2022). Mediating Effect of Sleep Quality on the Relationship Between Electronic Screen Media Use and Academic Performance Among College Students. Nat. Sci. Sleep.

[64] Sarvey D., Welsh J.W. (2021). Adolescent substance use: Challenges and opportunities related to COVID-19. J. Subst. Abus. Treat..

[65] Brackett M. (2019). Permission to Feel: Unlocking the Power of Emotions to Help Our Kids, Ourselves, and Our Society Thrive.

